# Alga-PrAS (Algal Protein Annotation Suite): A Database of Comprehensive Annotation in Algal Proteomes

**DOI:** 10.1093/pcp/pcw212

**Published:** 2017-01-06

**Authors:** Atsushi Kurotani, Yutaka Yamada, Tetsuya Sakurai

**Affiliations:** 1 RIKEN Center for Sustainable Resource Science, 1-7-22 Suehiro, Tsurumi, Yokohama, Kanagawa, 230-0045, Japan; 2 Interdisciplinary Science Unit, Multidisciplinary Science Cluster, Research and Education Faculty, Kochi University, 200 Otsu, Monobe, Nankoku, Kochi, 783-8502, Japan

**Keywords:** Algae, Comparative analysis, Database, Gene function, Protein properties

## Abstract

Algae are smaller organisms than land plants and offer clear advantages in research over terrestrial species in terms of rapid production, short generation time and varied commercial applications. Thus, studies investigating the practical development of effective algal production are important and will improve our understanding of both aquatic and terrestrial plants. In this study we estimated multiple physicochemical and secondary structural properties of protein sequences, the predicted presence of post-translational modification (PTM) sites, and subcellular localization using a total of 510,123 protein sequences from the proteomes of 31 algal and three plant species. Algal species were broadly selected from green and red algae, glaucophytes, oomycetes, diatoms and other microalgal groups. The results were deposited in the Algal Protein Annotation Suite database (Alga-PrAS; http://alga-pras.riken.jp/), which can be freely accessed online.

## Introduction

Algae are smaller organisms than land plants and offer clear advantages over terrestrial species for use in research in terms of rapid production, short generation time and varied commercial applications. Thus, algae are a very promising group of organisms for potential commercial applications, such as food and feed production, nutritional supplements, biofuel sources and environmental improvement through hydrogen production (Wijffels and Barbosa 2010, Draaisma et al. 2013, Torzillo et al. 2015). In the algal food and nutritional supplement sector, *Chlorella vulgaris* and *Spirulina platensis* have already been commercialized as health foods (Beheshtipour et al. 2013, Borowitzka 2013). However, while several studies in the biofuel sector have investigated selection, cultivation, extraction and purification of specific algal species and strains (Carvalho et al. 2006, Chisti 2007), a consensus has not yet been reached on costs and best practices in algal production (Passell et al. 2013, Medipally et al. 2015). Thus, studies investigating the development of practical and effective algal production techniques are important, and will improve our understanding of both aquatic and terrestrial plants, considering that algae are common ancestors of vascular plants (Reijnders et al. 2014, Bhattacharya et al. 2015).

The entire nuclear genome sequences of the red alga *Cyanidioschyzon merolae* (Matsuzaki et al. 2004) and the diatom *Thalassiosira pseudonana* (Armbrust et al. 2004) were determined. Subsequently, next-generation applications, including sequence assembly tools and gene prediction tools, have enabled the sequencing of algal species (Kim et al. 2014). As a result, over 30 whole algal genomes have been sequenced to date (Kim et al. 2014, Reijnders et al. 2014). These representative genomes, except for those of the two species mentioned above, include the green algae *Ostreococcus tauri* (Derelle et al. 2006) and *Chlamydomonas reinhardtii* (Merchant et al. 2007) of the Viridiplantae kingdom (including green plants), the red alga *Galdieria sulphuraria* (Schonknecht et al. 2013) and the glaucophyte *Cyanophora paradoxa* (Price et al. 2012). Additionally, genomes of the diatoms *Phaeodactylum tricornutum* (Chromista) (Bowler et al. 2008), *Aureococcus anophagefferens* (Pelagophyceae) (Gobler et al. 2011), *Ectocarpus siliculosus* (Phaeophyceae) (Cock et al. 2010), *Emiliania huxleyi* (Haptophyceae) (Read et al. 2013) and *Guillardia theta* (Cryptophyceae) (Curtis et al. 2012) are also included.

There is a considerable amount of information about land plants based on genomic, transcriptomic, proteomic and metabolomic analyses. The land plant *Arabidopsis thaliana* is currently one of the most commonly used experimental plants, as it has a small genome and a short life cycle. Information on *Arabidopsis* research was organized into The Arabidopsis Information Resource (TAIR) (Berardini et al. 2015). Similarly, *Oryza sativa*, also a well-studied species, is one of the most important crop plant models. Information regarding the genome and functional gene annotations in *O. sativa* is housed in the Michigan State University Rice Genome Annotation Project database (MSU Rice) (Ouyang et al. 2007) and the Rice Annotation Project database (RAP-DB) (Sakai et al. 2013). Furthermore, the genomic sequence information of various plant species has been updated in the JGI Genome Portal (Nordberg et al. 2014), Phytozome (Goodstein et al. 2012), GRAMENE (Youens-Clark et al. 2011) and PlantGDB (Dong et al. 2004). Moreover, in order to promote the development of functional annotation of genes in plants, several approaches and databases have been developed, accruing information on the transcriptome or metabolome in plants, as follows: transcription factor (TF) annotation at both family and gene levels (PlantTFDB) (Guo et al. 2008), TF integration of gene expression data for plants (ATTED-II) (Aoki et al. 2016b), integrative analysis for plant hormone accumulation and gene expression in rice (UniVIO) (Kudo et al. 2013), and utilization of transcriptomic and metabolic profiles among plant tissues (PRIMe Update) (Sakurai et al. 2013). These databases can be used to study gene function. Several large-scale experimental and computational approaches have also been adopted to enhance the study of functional annotation in plant proteomes (Kourmpetis et al. 2011, Akiyama et al. 2014, Clemente and Jamet 2015, Kurotani et al. 2015).

In algae, many general resources and culture collection databases exist, including: AlgaTerra (http://www.algaterra.org), AlgaeBase (http://www.algaebase.org) (Guiry et al. 2014), SAG (http://www.uni-goettingen.de/en/184982.html), NIES (http://mcc.nies.go.jp), and KU-MACC (http://www.research.kobe-u.ac.jp/rcis-ku-macc/E.index.html). Concomitantly, molecular-based biological approaches to algae have also been systematically recorded and made available through databases. These are: the database of genomic information of photosynthesis (Pico-PLAZA) (Vandepoele et al. 2013), the database of algal gene expression (ALCOdb) (Aoki et al. 2016a), the Marine Microbial Eukaryote Transcriptome Sequencing Project (MMETSP) (Keeling et al. 2014), the database of *Pleurochrysis* transcripts (Pleurochrysome) (Yamamoto et al. 2016), the database of algal metabolic pathways (ALGAEpath) (Zheng et al. 2014) and the metabolome analyses of *Cyanidioschyzon merolae* (Sumiya et al. 2015). Although biological information on algae has been steadily increasing through research, it is still insufficient to comprehensively understand the functional annotations of algal genes.

*Chlamydomonas reinhardtii* is one of the best-studied green algae of recent years (May et al. 2009, Blaby et al. 2014, Aoki et al. 2016). According to the UniProt database (Bateman et al. 2015), as of July 2016 there were 14,716 records of *C. reinhardtii.* However, two-thirds of these records (9,860 records) are not informative annotations (e.g. ‘Predicted protein’, ‘Predicted protein -Fragment-’, and ‘Uncharacterized protein’) and only a subset of fewer than 50 annotations have experimentally validated functions (Reijnders et al. 2014). Therefore, comprehensive algal proteome information is far from satisfactory. Here we report the development of the Algal Protein Annotation Suite (Alga-PrAS) database, a user-friendly website with algal proteome information, specifically physicochemical, structural and functional annotations of algal proteome data.

## Results and Discussion

### Protein sequence sets

To provide unbiased proteome information, we prepared non-redundant protein sequence sets from whole-protein sequence sets of 34 species as follows. Sequences with fewer than 50 amino acids were omitted as these short sequences typically define peptides (Orlowski and Bujnicki 2008, Saghatelianr and Couso 2015). To avoid calculation failure of analytic tools, such as DIpro (Cheng et al. 2006), SSpro (Cheng et al. 2005) and DROP (Ebina et al. 2011), we removed sequences with more than 4,000 amino acids. Redundant sequences were removed by individually clustering protein sequences of each species. This was performed with the CD-HIT program (Fu et al. 2012) with default runtime options. Finally, 34 non-redundant protein sequence sets were independently obtained, totaling 510,123 sequences (Supplementary Table S1).

### Annotation of algal proteomes by sequence similarity against public databases

Nonredundant algal protein sequences were aligned with BLASTP (Altschul et al. 1997, Altschul et al. 2005) against UniProtKB (Bateman et al. 2015). As a result, 46.2% of the algal protein sequences could achieve a hit with an e-value lower than 1e−10 (Table 1). The hit sequence percentages of 14 algae did not reach 50% (Supplementary Table S2). Approximately 60% of the algal proteins were annotated successfully, even when all assignment results to public databases were totaled. These results imply that functional genomic investigations are less efficient in algae than in land plants. Therefore, in addition to sequence similarity, the functional annotation of algal genomes should be enhanced by analytic approaches that employ structural and physicochemical properties, and post-translational modification (PTMs).

**Table 1:** Percentages of sequences annotated by the KOG, Pfam, UniProtKB, GO and PDB databases

Class	Percentage of annotated sequences^a^ (%)					
	KOG	Pfam	UniProtKB	GO	PDB	Total^b^ (%)
Land plants	34.2	67.9	70.7	44.9	47.4	77.3
Algae	26.9	54.6	46.1	34.7	36.6	60.3
Green algae	31.8	60.7	55.9	38.9	41.7	67.3
Red algae	34.6	61.5	55.7	41.0	44.0	67.1
Glaucophyceae	14.0	31.4	25.7	19.5	19.8	37.0
Oomycetes	28.6	57.8	49.6	37.8	38.5	64.2
Diatoms	25.1	53.7	39.8	33.7	34.2	57.9
Other microalgae	22.8	50.8	39.1	31.2	33.4	56.0
All species	28.0	56.5	49.6	36.2	38.2	62.8

^a^ Poor annotations such as ‘poorly characterized’ in KOG, ‘domain unknown function (DUF)’ in Pfam, and ‘Uncharacterized protein,’ ‘Putative uncharacterized,’ ‘Unnamed product’ and only ID in UniProtKB, were excluded from hits.

^b^ Values were calculated by combining the results of KOG, Pfam, UniProtKB, GO and PDB.

### Protein property information of Alga-PrAS

Compared with higher organisms, such as *Homo sapiens* (Imanishi et al. 2004), *Mus musculus* (McGarvey et al. 2015) and *Arabidopsis* (Berardini et al. 2015), available information and tools for the comprehensive annotation of algal proteomes are scarce. Therefore, it is important to provide information on algal protein function, specially that relating to protein properties. Physicochemical properties are useful to understand fundamental aspects of the structural stability, reactivity and solubility of proteins. Structural properties aid in identifying protein secondary structure and functional annotations against other existing protein sequences that are assigned to structural and functional domains or regions. In addition, PTM and subcellular localization aid in elucidating potential protein diversity, structure and function. We estimated 28 protein properties to improve the information on algal protein function with respect to various protein properties as stated above (Table 2). All information on the protein properties was integrated and housed in the Alga-PrAS database.

**Table 2:** List of calculated protein properties in this study

Classification of protein properties	Sub-classification of protein properties
Physicochemical properties	Protein length
	Percentage of charged residues
	Percentage of nonpolar residues
	Percentage of acidic residues
	Percentage of basic residues
	Grand average value of hydropathicity index (GRAVY)
	Isoelectric point (pI)
	Probability of protein solubility
Structural properties	Percentage of beta-pleated sheet secondary structure
	Percentage of disordered residues
	Number of long disordered regions
	Existence of signal peptide cleavage site
	Number of transmembrane helices
	Number of S–S bonds
	Number of domain linkers
	Number of internal repeats
	Number of PEST regions
Post-translational modifications (PTMs) and subcellular localization	Number of Ser, Thr and Tyr phosphorylation sites
	Number of O-linked glycosylation sites
	Number of N-linked glycosylation sites
	Number of ubiquitination sites
	Protein subcellular localization sites

### Search interface of Alga-PrAS

We developed a proteome annotation database, Alga-PrAS, which includes an enormous amount of proteome data (over 500,000 protein sequences of 34 species in total) and is available via the web interface at http://alga-pras.riken.jp/. To obtain protein information from the Alga-PrAS database, four search functions—Property Search, Identifier (ID) Search, Keyword Search and Sequence Search—are provided in the Alga-PrAS database. These are detailed below.

#### Property Search

**Fig. 1:**
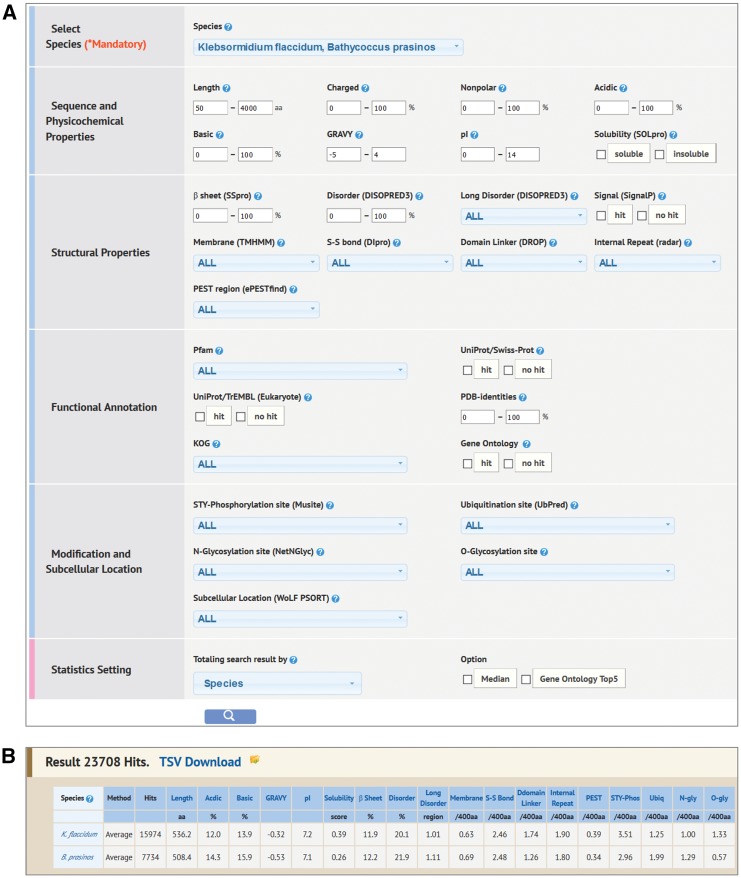
Property Search interface. (A) Users can search by multiple protein properties on the Property Search page. (B) Example of a summary table from the Property Search results.

**Fig. 2:**
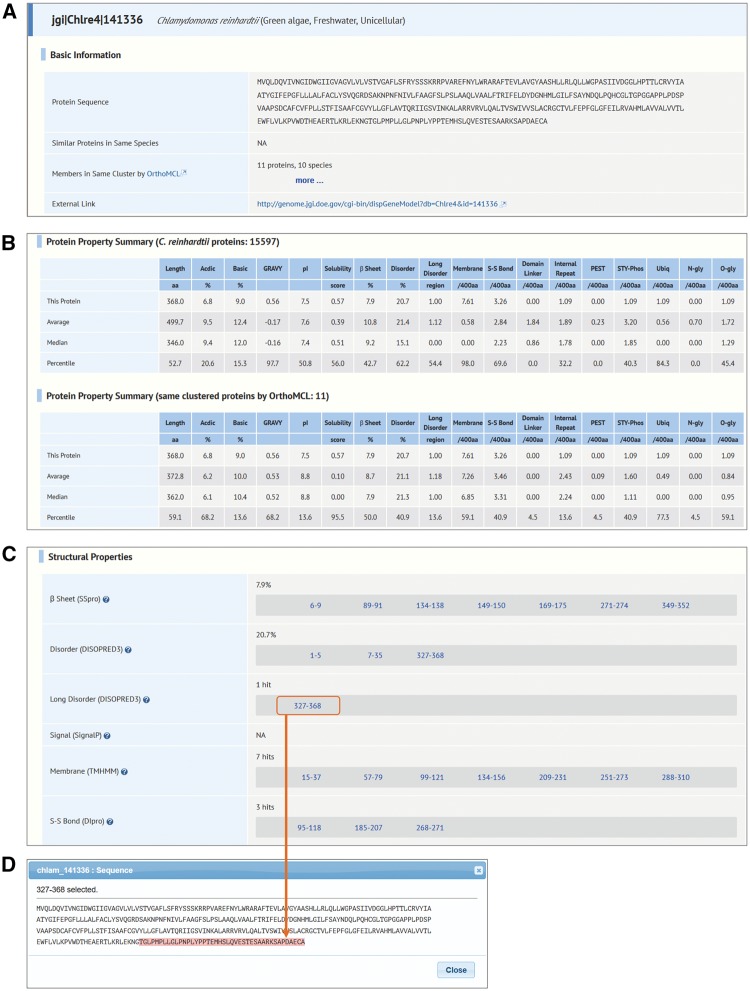
Typical examples of annotation detail page. (A) Basic information on a protein in Alga-PrAS. (B) Summary with average, median and percentile values in relation to proteins from identical species (upper portion) and identical clustered proteins by OrthoMCL (lower portion). (C) Structural properties. (D) Sequence window for highlighting position data for regions or sites.

Property Search is the most comprehensive search function for accessing Alga-PrAS data. It provides a search function from 28 protein properties against 34 species proteomes (Fig. 1A). On the results page, a summary of the searched data containing average or median values for each property is shown in a summary statistics table (Fig. 1B). Subsequently, when users click on one of the hyperlinked items (e.g. species, taxonomic class) on the left side of the table, IDs belonging to the selected items are listed on the same page. The listed IDs are linked to the annotation detail page of each protein (Fig. 2). In this search there is also a convenient function for comparison analysis among the Alga-PrAS data. By setting the display option, the summary statistics table can be sorted by species, taxonomic classification, habitat, unicellularity or multicellularity, protein cluster and KOG, meaning that biological species can be selected by users based on common classification terms (land plants, green algae, red algae, Glaucophyceae, oomycetes, diatoms and other microalgae), habitat (freshwater, marine, terrestrial and ubiquitous), whether an organism is composed of one or multiple cells, species-specific or common protein clusters by orthologous clustering with the OrthoMCL tool (single-species cluster, all-species cluster and other) (Fischer et al. 2011), or 25 KOG function categories (Koonin et al. 2004) (Supplementary Tables S1 and S3). In addition, to visualize numeric data the user can click a property item in the summary statistics table and display a bar chart frame.

#### ID Search and Keyword Search

**Fig. 3:**
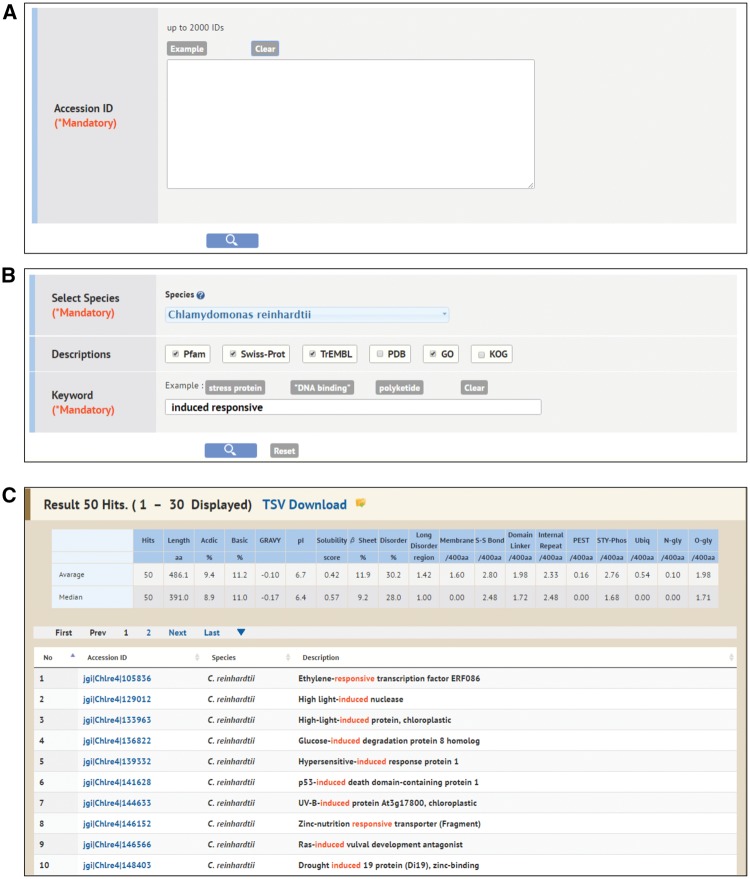
Interfaces of ID Search and Keyword Search. (A) ID Search. This provides a search function by inputting arbitrary IDs in the text box as a query. (B) Keyword Search. This is an annotation search function against the assigned descriptions of the public databases. (C) Example of the results of Keyword Search. The example is the search result for the species *Chlamydomonas reinhardtii*, the description *Pfam*, *Swiss-Prot* and *TrEMBL*, and the keywords *induced responsive*.

ID Search is a simpler search function for accessing the Alga-PrAS data if the user knows the accession IDs of proteins on public protein databases such as UniProtKB and Pfam. It provides a search function by inputting arbitrary IDs in the text box as a query (Fig. 3A). Keyword Search is an annotation search function against the assigned descriptions of the Pfam, UniProt/Swiss-Prot, UniProt/TrEMBL, PDB, GO and KOG databases housed in advance in Alga-PrAS (Fig. 3B). A multiple keyword search is performed when the introduced keywords are separated by spaces. In addition, an exact phrase search is performed by enclosing keywords within quotation marks, and, to exclude specific words, users can use a hyphen as a prefix for the keyword they wish to exclude. For example, using (Myb -like) as a search keyword excludes the word ‘like’ from the search results. The ID list from the ID or Keyword Search is shown on the results page (Fig. 3C). Listed IDs are linked to the annotation detail page of each protein in the same manner as that of Property Search (Fig. 2).

#### Sequence Search

**Fig. 4:**
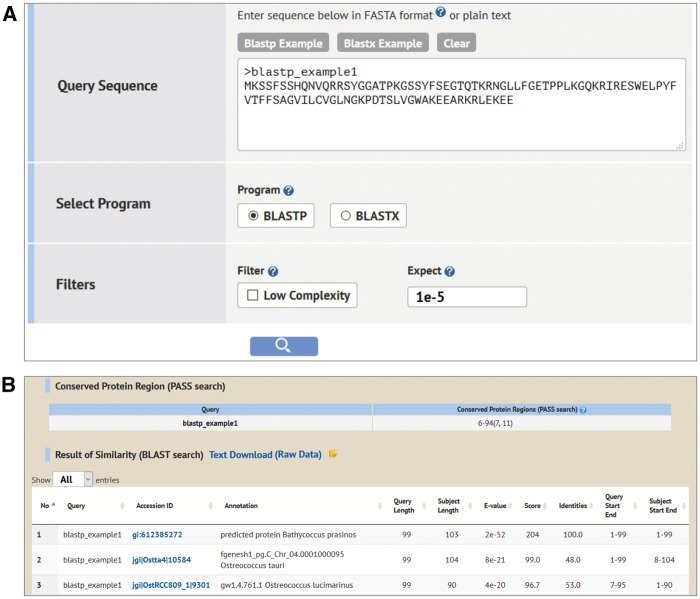
Sequence Search interface. (A) Sequence Search allows protein or nucleic acid sequences to be submitted in the FASTA format as a query with the option of a cutoff e-value. (B) Example of Sequence Search results. The result tables for BLAST and PASS searches show that the conserved protein region is located from six to 94 amino acids of the query protein sequence.

Sequence Search contains two search processes for algal data with users’ arbitrary sequences (Fig. 4A). One is a BLAST (Altschul et al. 1997, Altschul et al. 2005) search against the algal sequences in Alga-PrAS. The other is a conserved protein region search with the PASS tool (Kuroda et al. 2000), which determines the N-terminal site and the C-terminal site of conserved protein regions among diverse organisms using the BLAST result. Therefore, users can confirm the information on sequence similarity and conserved protein regions between their arbitrary sequences and the algal sequences housed in Alga-PrAS. This search allows protein or nucleic acid sequences to be submitted in the FASTA format as a query, with the option of a cutoff e-value. The result tables for BLAST and PASS searches are shown in the footer of the same page (Fig. 4B). The searched IDs are linked to the annotation detail page of each protein in the same manner as for the results page of the search functions mentioned above (Fig. 2).

### Annotation detail page

The annotation detail page displays all the information available for an individual protein. The basic information, including amino acid sequence, IDs of similar proteins omitted in the clustering process by the CD-HIT tool in order to remove redundant sequences, and the IDs in the same cluster of all protein sequences in Alga-PrAS by the OrthoMCL tool, is displayed in the top part of page (Fig. 2A). Next, the summary tables of protein properties for proteomes of identical species and clustered proteins are displayed under the basic information section (Fig. 2B). Items in the summary tables consist of average and median values and percentile ranks for each protein property. Thus, the status of the query protein can be easily recognized among the Alga-PrAS data. Finally, all protein properties from sequence analyses are displayed under the summary tables (Fig. 2C). When users click the hyperlinked position data for regions or sites, these are highlighted on the protein sequence in an additional window (Fig. 2D). Additionally, external links to protein sequences and annotations (Pfam, UniProtKB, PDB, KOG and GO databases) are provided to enable verification with the original information on the resource websites.

### Download

Users can download all information at the resources page in Alga-PrAS (http://alga-pras.riken.jp/menta.cgi/algapras/resources). In addition to the bulk download page, search results can also be downloaded as a tab-separated value (TSV) file each time a search is performed.

### Examples of utilization of Alga-PrAS

#### Exploring candidate G protein-coupled receptors (GPCRs)

**Fig. 5:**
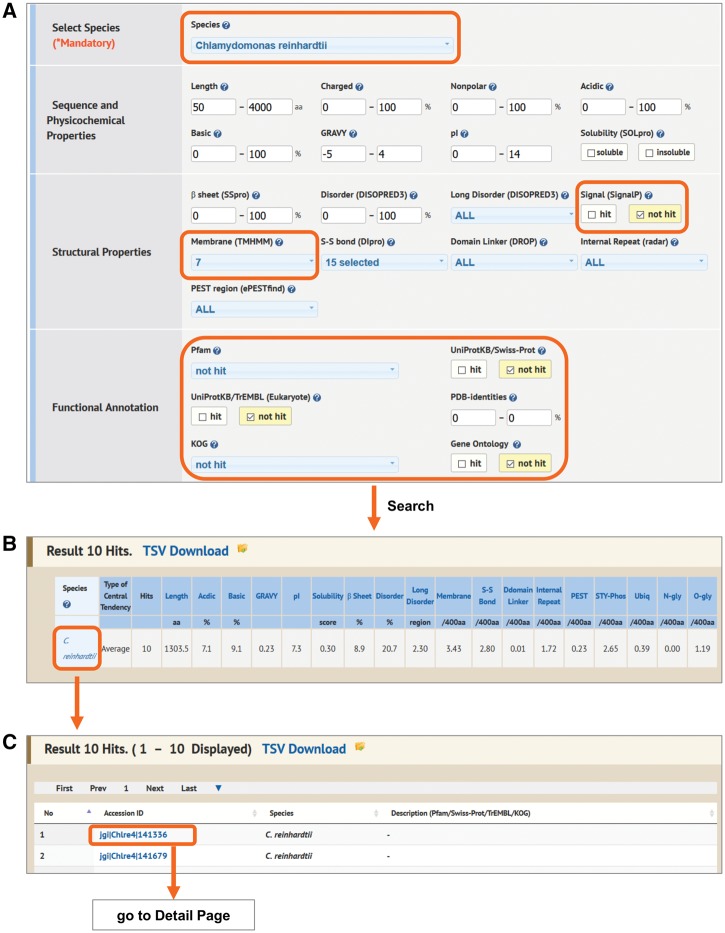
Search example of the exploration of candidates of G protein-coupled receptors (GPCRs). The settings for Property Search are as follows; ‘*Chlamydomonas reinhardtii*’ in the Species field (e.g. ‘7’ in Membrane), ‘not hit’ in Signal, Pfam, UniProtKB/Swiss-Prot, UniProtKB/TrEMBL, KOG and Gene Ontology, and ‘0%’ in PDB (A). The results identified 10 protein sequences as candidate GPCRs (B). Users click ‘C. reinhardtii’ on the Species column on the summary table, and the accession IDs which are searched by the above process are then displayed (C).

GPCRs constitute a large and diverse family of proteins that regulate various cellular functions involved in physiological responses (Guan et al. 1992, Pierce et al. 2002). We explored GPCR candidates in *C. reinhardtii* protein sequences known to contain seven membrane helix domain receptors and to lack a cleavable signal sequence (Singer 1990, Guan et al. 1992). First, we set ‘*Chlamydomonas reinhardtii*’ in the Species field (e.g. ‘7’ in Membrane, ‘not hit’ in Signal, Pfam, UniProtKB/Swiss-Prot, UniProtKB/TrEMBL, KOG and Gene Ontology, and ‘0%’ in PDB on the Property Search page; Fig. 5A). Negative settings in Pfam to PDB were intended to retrieve proteins that do not have functional annotations in these databases. This approach identified 10 protein sequences as candidate GPCRs (Fig. 5B). Next, we click on ‘*C. reinhardtii*’ in the Species column on the summary table; the accession IDs retrieved as a result of the above search process are displayed (Fig. 5C). When one of the protein IDs (e.g. jgi|Chlre4|141336) is clicked, the annotation detail page of the protein is displayed (Fig. 2). In this page the following information is shown: (i) other proteins belonging to the same cluster in the ‘Members in same cluster by OrthoMCL’ field in basic information (Fig. 2A), and (ii) the summary statistics of protein properties in the *C. reinhardtii* proteome and of the members of the same cluster (Fig. 2B) in the protein properties and the structural properties (Fig. 2C).

#### Number of PTMs in species-specific and common protein clusters in proteomes of land plants and algae

It is reported that the conservation of protein structure regions has been associated with higher amino acid substitution rates and faster evolution (Kim et al. 2008, Mosca et al. 2012, Brunquell et al. 2014). Thus, differences in the number of PTMs between species-specific protein clusters and common protein clusters of algae proteomes may be expected. To explore this in Alga-PrAS, protein clusters for all the proteins used in this study were created using the OrthoMCL tool (Fischer et al. 2011), housing in advance the results in the Alga-PrAS database as described previously. Then, protein clusters consisting of all 34 species used in this study were defined as common protein clusters. Protein clusters involving only one species were regarded as species-specific protein clusters. The content of all PTM parameters, including phosphorylation, glycosylation and ubiquitination in species-specific protein clusters or in common protein clusters of each taxonomic class, is shown in Table 3. In this analysis, we normalized the number of PTM sites to the same length (400 amino acids) based on the dataset’s average protein length. Information regarding PTM parameters can be obtained from the bulk download file. The contents of phosphorylation parameters were 1.1–5.4 times higher in species-specific protein clusters than in common protein clusters and the occurrence of phosphorylation in ratios of species-specific/common protein clusters in algal species was higher than in land plants (Table 3). This result may imply that algal species, which are simpler than land plants, utilize phosphorylation better than land plants. To date, many studies have been conducted on plant protein phosphorylation sites on photosynthetic membranes, and under a variety of conditions from biotic and abiotic stresses to changing nutrient environments. The principle of activation and inactivation of proteins by phosphorylation and the function of phosphorylated amino acid residues as docking sites have also been well characterized in the field of plant signal transduction (Turkina et al. 2006, Turkina and Vener 2007, Camoni et al. 2000, Nakagami et al. 2010).

**Table 3:** Preference of protein disorder and PTMs in species-specific protein clusters and common protein clusters for each taxonomic class

Taxonomic class		Disorder	S-pho/400aa^d^	T-pho/400aa^d^	Y-pho/400aa^d^	O-gly/400aa^d^	N-gly/400aa^d^	Ubi/400aa^d^
Land plants	Specific^a^	16%	1.3	0.5	0.5	0.9	1.3	1.1
	Common^b^	13%	0.7	0.3	0.4	0.6	1.2	0.7
	S/C ratio^c^	1.2	2.0	1.7	1.1	1.4	1.1	1.5
Green algae	Specific	20%	2.4	1.2	0.6	1.8	0.9	0.9
	Common	12%	0.6	0.3	0.5	0.8	0.9	0.6
	S/C ratio	1.7	4.0	3.6	1.4	2.4	0.9	1.6
Red algae	Specific	12%	1.7	0.9	0.6	1.4	1.0	0.9
	Common	14%	0.7	0.4	0.5	0.8	1.0	0.6
	S/C ratio	0.9	2.3	2.1	1.3	1.7	1.0	1.5
Glaucophyceae	Specific	14%	2.3	1.0	0.5	1.8	0.8	0.8
	Common	10%	0.5	0.3	0.3	0.9	1.0	0.6
	S/C ratio	1.4	4.9	3.6	1.5	2.0	0.8	1.4
Oomycetes	Specific	14%	1.3	0.7	0.6	0.9	1.3	0.8
	Common	12%	0.6	0.3	0.4	0.7	1.1	0.7
	S/C ratio	1.1	2.3	2.2	1.3	1.4	1.1	1.2
Diatoms	Specific	20%	1.8	0.8	0.6	1.0	2.1	1.8
	Common	10%	0.3	0.2	0.4	0.6	1.2	0.7
	S/C ratio	2.0	5.4	4.7	1.7	1.9	1.7	2.7
Other microalgae	Specific	16%	2.1	0.9	0.6	1.2	1.0	1.4
	Common	11%	0.7	0.4	0.4	0.8	0.9	0.7
	S/C ratio	1.4	3.1	2.7	1.4	1.6	1.1	2.1

^a^ The Specific category (species-specific protein clusters) involves just one species in a cluster using the OrthoMCL tool.

^b^ The Common category (common protein clusters) involves all 34 species used in this study.

^c^ Ratio of specific to common values.

^d^ Average of normalized value of predicted PTM sites. The number of predicted PTM sites was normalized per 400 amino acids (aa).

## Conclusion

Alga-PrAS is the most comprehensive resource for integrating abundant algal proteome information, and has an effective interface to enable the interpretation of algal proteome features. Importantly, the system can be expected to enhance gene functional annotation and further developments in algal species.

## Materials and Methods

### Resources for protein sequences

In this study we used 31 algal proteome sequence sets involving 12 green algae, five red algae, one Glaucophyceae, four oomycetes, three diatoms and six other algal species (Table 4). Three land plant species, *Arabidopsis thaliana* (Swarbreck et al. 2008), *Selaginella moellendorffii* (Banks et al. 2011) and *Physcomitrella patens* (Rensing et al. 2008) were also used (Table 4). Non-redundant protein sequence sets were prepared. First, sequences of less than 50 and more than 4,000 amino acids were excluded to avoid calculation failure in the prediction processes performed with DIpro (Cheng et al. 2006), SSpro (Cheng et al. 2005) and DROP (Ebina et al. 2011). To prepare non-redundant proteome sequence sets of each species, individual protein clusters of each species were created with the CD-HIT program (Fu et al. 2012) with default runtime parameters, and a protein sequence set specific to each species was used as input data..2

**Table 4:** List of protein sequence resources in this study

Classification	Species	Proteome resources	References for genomic analysis
Green algae	*Klebsormidium flaccidum*	*Klebsormidium flaccidum* Genome Project^g^	Hori et al. 2014
	*Ostreococcus lucimarinus*	JGI Genome Portal^h^	Palenik et al. 2007
	*Ostreococcus tauri*	JGI Genome Portal^h^	Derelle, et al. 2006
	*Micromonas pusilla*	JGI Genome Portal^h^	Worden et al. 2009
	*Micromonas sp. RCC299*	JGI Genome Portal^h^	Worden, et al. 2009
	*Bathycoccus prasinos*	NCBI^i^	Moreau et al. 2012
	*Volvox carteri*	JGI Genome Portal^h^	Prochnik et al. 2010
	*Chlamydomonas reinhardtii*	JGI Genome Portal^h^	Merchant, et al. 2007
	*Monoraphidium neglectum*	NCBI^i^	Bogen et al. 2013
	*Coccomyxa subellipsoidea*	JGI Genome Portal^h^	Blanc et al. 2010
	*Chlorella variabilis*	JGI Genome Portal^h^	Blanc, et al. 2010
	*Auxenochlorella protothecoides*	NCBI^i^	Gao et al. 2014
Red algae	*Cyanidioschyzon merolae*	*Cyanidioschyzon merolae* Genome Project^j^	Matsuzaki, et al. 2004, Nozaki et al. 2007
	*Galdieria sulphuraria*	NCBI^i^	Schonknecht, et al. 2013
	*Pyropia yezoensis*	NRIFS^k^	Nakamura et al. 2013
	*Chondrus crispus*	NCBI^i^	Collen et al. 2013
	*Porphyridium purpureum*	*Porphyridium purpureum* Genome Project^l^	Bhattacharya et al. 2013
Glaucophyceae	*Cyanophora paradoxa*	*Cyanophora* Genome Project^m^	Price et al. 2012
Oomycetes	*Phytophthora ramorum*	JGI Genome Portal^h^	Tyler et al. 2006
	*Phytophthora sojae*	JGI Genome Portal^h^	Tyler et al. 2006
	*Phytophthora infestans*	Superfamily database^n^	Haas et al. 2009
	*Phytophthora capsici*	JGI Genome Portal^h^	Lamour et al. 2012
Diatoms	*Phaeodactylum tricornutum*	JGI Genome Portal^h^	Bowler et al. 2008
	*Fragilariopsis cylindrus* sp. CCMP1102	JGI Genome Portal^h^	http://genome.jgi.doe.gov/Fracy1/Fracy1.info.html
	*Thalassiosira pseudonana*	JGI Genome Portal^h^	Armbrust et al. 2004
Other algal species	*Aureococcus anophagefferens^a^*	JGI Genome Portal^h^	Gobler et al. 2011
	*Ectocarpus siliculosus^b^*	JGI Genome Portal^h^	Cock et al. 2010
	*Symbiodinium minutum^c^*	OIST^o^	Shoguchi et al. 2013
	*Emiliania huxleyi^d^*	NCBI^i^	Read et al. 2013
	*Guillardia theta^e^*	NCBI^i^	Curtis et al. 2012
	*Bigelowiella natans^f^*	JGI Genome Portal^h^	Curtis et al. 2012
Land plants	*Arabidopsis thaliana*	TAIR^p^	Swarbreck et al. 2008
	*Selaginella moellendorffii*	JGI Genome Portal^h^	Banks et al. 2011
	*Physcomitrella patens*	JGI Genome Portal^h^	Rensing et al. 2008

^a–f^ Other algal species (*Aureococcus anophagefferens*, *Ectocarpus siliculosus*, *Symbiodinium minutum*, *Emiliania huxleyi*, *Guillardia theta* and *Bigelowiella natans*) belong to Pelagophyceae, Phaeophyceae, Dinophyceae, Haptophyceae, Cryptophyceae and Chlorarachniophyceae, respectively.

^g^ http://www.plantmorphogenesis.bio.titech.ac.jp/∼algae_genome_project/klebsormidium/index.html (Hori et al. 2014).

^h^ http://genome.jgi.doe.gov (Nordberg et al. 2014).

^i^ http://www.ncbi.nlm.nih.gov (Pruitt et al. 2007, Pruitt et al. 2012).

^j^ http://merolae.biol.s.u-tokyo.ac.jp (Matsuzaki et al. 2004).

^k^ http://nrifs.fra.affrc.go.jp/ResearchCenter/5_AG/genomes/nori/index.html (Nakamura et al. 2013).

^l^ http://cyanophora.rutgers.edu/porphyridium (Bhattacharya et al. 2013).

^m^ http://cyanophora.rutgers.edu/cyanophora/home.php (Price et al. 2012).

^n^ http://supfam.org/SUPERFAMILY (Oates et al. 2015).

^o^ http://marinegenomics.oist.jp/symb/viewer/info?project_id=21 (Shoguchi et al. 2013).

^p^ https://www.arabidopsis.org (Swarbreck et al. 2008).

### Calculation of protein properties

#### Physicochemical properties

The percentages of acidic, basic, charged and non-polar amino acids, as well as protein length and isoelectric point (pI), were calculated using the ProteoMix tool (Chikayama et al. 2004). The GRAVY index was calculated with the GRAVY algorithm (Kyte and Doolittle 1982). Protein solubility was determined using the SOLpro tool (Magnan et al. 2009).

#### Secondary structural properties

To detect protein properties related to secondary structure, we used the following tools: SignalP4.0 (Petersen et al. 2011), TMHMM2.0 (Krogh et al. 2001), DROP (Ebina et al. 2011), DIpro2.0 (Cheng et al. 2006), SSpro4 (Cheng et al. 2005), RADAR (Heger and Holm 2000), DISOPRED3 (Jones and Cozzetto 2015) and ePESTfind of EMBOSS (Rogers et al. 1986, Rice et al. 2000) to determine the presence of signal peptides, transmembrane helix domains, interdomain linkers, S–S bonds, secondary structures, internal repeats, intrinsically disordered regions and PEST regions, respectively.

#### Functional and structural annotations

We assigned protein annotations of KOG (Tatusov et al. 2000), UniProt/Swiss-Prot (Boutet et al. 2016), UniProtKB/TrEMBL (eukaryote) (Bateman et al. 2015) and PDB (Westbrook et al. 2003, Berman et al. 2014) using the BLASTP program with an e-value lower than 1e−10. The Pfam (Finn et al. 2016) and GO terms (Blake et al. 2015) were detected using InterProScan5 software (Hunter et al. 2012).

#### Modification and subcellular localization

To infer PTM and subcellular localization, we used the following tools and algorithms. Serine (Ser; S), threonine (Thr; T) or tyrosine (Tyr; Y) phosphorylation sites were detected with Musite1.0.1 (Gao et al. 2010) with the database option of Eukaryote-General-Ser-Thr;Eukaryote-General-Tyr. *O*-glycosylation sites were detected based on Gomord’s algorithm (Gomord et al. 2010). *N*-glycosylation sites were detected by combining the results of the NetNglyc1.0 tool (http://www.cbs.dtu.dk/services/NetNGlyc) with the signal peptide (SignalP) option and the TMHMM2.0 tool. Thus, we detected extracellular *N*-glycosylation sites with TMHMM2.0, and the number of signal peptides in the sequence was calculated with SignalP from NetNglyc1.0 to remove false-positive data with NetNglyc1.0. Ubiquitination sites were detected with the UbPred tool (Radivojac et al. 2010) with a medium confidence option. Transmembrane helix regions were detected with the TMHMM2.0 tool. Subcellular localizations were detected with the WoLF PSORT tool (Horton et al. 2007). Additionally, for the protein sequences of the diatoms *Fragilariopsis cylindrus* (CCMP 1102), *Phaeodactylum tricornutum* and *Thalassiosira pseudonana*, the cryptophyte *Guillardia theta* and the dinoflagellate *Symbiodinium minutum*, we used the HECTAR tool (Gschloessl et al. 2008) because the chloroplasts of these five algal species evolved from secondary endosymbiosis (Gruber et al. 2015).

### Classification of species-specific and common protein clusters

To determine the number of PTMs in species-specific and common protein clusters in proteomes of land plants and algae, we created protein clusters among all the protein sequences in this study. First, we calculated pairwise sequence similarities between all the protein sequences by using the BLASTP program with an e-value lower than 1e−5. Subsequently, protein clusters were estimated by the Markov Clustering (MCL) algorithm employed in OrthoMCL1.4 (Fischer et al. 2011) with the BLASTP results and the default runtime parameters. Finally, a singlet and a cluster consisting of only one species were classified as a species-specific protein, and a cluster consisting of all 34 species was classified as a common protein cluster.

### System availability and implementation

Alga-PrAS was implemented in the Linux operating system (CentOS 6.8, 64 bit) with a MENTA web application framework based on Perl 5.1.0 and MySQL 5.7.13 as a database engine, and tested on the following web browsers: Microsoft Edge 25, Internet Explorer 10+, Google Chrome 51+ and Firefox 41+.

## Supplementary Data

Supplementary data are available at PCP online.

## Supplementary Material

Supplementary Data

## Abbreviations

Alga-PrAS: Algal Protein Annotation Suite
BLAST: Basic Local Alignment Search Tool
GRAVY: grand average value of hydropathicity index
IDR: intrinsically disordered region
NCBI: National Center for Biotechnology Information
PTM: post-translational modification
