# Supplementary material for: Alga-PrAS (Algal Protein Annotation Suite): A Database of Comprehensive Annotation in Algal Proteomes
Source: Plant Cell Physiol. 2017 Jan 6;58(1):e6. doi: 10.1093/pcp/pcw212 (PMC5444574; doi:10.1093/pcp/pcw212)
Supplement: Supplementary Data [file pcw212_Supp.pdf]

Supplementary information

Table S1. Number of protein sequences, average length of protein sequences, habitat, and multicellularity in the analyzed proteomes

Taxonomic class	Species	Number of sequences		Average of protein sequence length after filtering	Habitat	Unicellular/Multicellular
		before filtering	after filtering			
Land plants	*Arabidopsis thaliana*	35386	26947	414	Terrestrial	Multicellular
	Physcomitrella patens	42392	25775	392	Terrestrial	Multicellular
	Selaginella moellendorffii	22285	20225	386	Terrestrial	Multicellular
Green algae	*Klebsormidium flaccidum*	16215	15974	536	Freshwater	Multicellular
	Ostreococcus lucimarinus	7773	7688	380	Marine	Unicellular
	Ostreococcus tauri	7725	7602	385	Marine	Unicellular
	Micromonas sp. RCC299	10103	9969	471	Marine	Unicellular
	Micromonas pusilla	10660	10557	441	Marine	Unicellular
	Bathycoccus prasinos	7892	7734	508	Marine	Unicellular
	Coccomyxa subellipsoidea C-169	9629	9374	425	Freshwater	Unicellular
	Chlorella variabilis	9994	9783	403	Freshwater	Unicellular
	Volvox carteri	14542	13925	569	Freshwater	Unicellular

	Chlamydomonas reinhardtii	16706	15597	500	Freshwater	Unicellular
	Auxenochlorella protothecoides	6927	6628	396	Freshwater	Unicellular
	Monoraphidium neglectum	16755	14128	367	Freshwater	Unicellular
Red algae	*Cyanidioschyzon merolae*	5014	4642	516	Freshwater	Unicellular
	Galdieria sulphuraria	7375	6371	419	Freshwater	Unicellular
	Pyropia yezoensis	10327	9731	292	Marine	Multicellular
	Chondrus crispus	9836	8331	380	Marine	Multicellular
	Porphyridium purpureum	8355	8202	476	Ubiquitous	Unicellular
Glaucophyceae	*Cyanophora paradoxa*	32167	31147	230	Freshwater	Unicellular
Oomycetes	*Phytophthora ramorum*	15743	13202	505	Terrestrial	Unicellular
	Phytophthora sojae	19027	16833	517	Terrestrial	Unicellular
	Phytophthora infestans	22658	19455	407	Terrestrial	Unicellular
	Phytophthora capsici	17414	16168	388	Terrestrial	Unicellular
Diatoms	*Phaeodactylum tricornutum*	10402	10063	460	Marine	Unicellular
	Fragilariopsis cylindrus CCMP 1102	27143	20616	429	Marine	Unicellular
	Thalassiosira pseudonana	11776	11484	492	Marine	Unicellular

Other microalgae	*Aureococcus anophagefferens*	11501	10772	519	Marine	Unicellular
	Ectocarpus siliculosus	16533	15577	519	Marine	Multicellular
	Symbiodinium minutum	47014	42522	555	Marine	Unicellular
	Emiliania huxleyi	38694	21992	401	Marine	Unicellular
	Guillardia theta	25457	19964	432	Marine	Unicellular
	Bigelowiella natans	21706	21145	436	Marine	Unicellular
	Total	593126	510123	437		

The filtering method is described in “Protein sequence sets” of the “Results and Discussions” section.

The habitat information is retrieved from AlgaeBase (http://www.algaebase.org/).

Unicellular/Multicellular information is referred in each original paper.

Table S2. Protein annotations by KOG, Pfam, and UniProtKB[a]

Taxonomic class	Species	Percentage of annotated sequences[a] (%)					
		KOG	Pfam	UniProtKB	GO	PDB	Total[b]
Land plants	*Arabidopsis thaliana*	37.4	74.7	86.2	50.8	49.8	89.0
	Physcomitrella patens	32.1	61.0	59.2	40.9	43.0	67.5
	Selaginella moellendorffii	32.5	67.8	64.7	42.0	49.9	74.2
Green algae	*Klebsormidium flaccidum*	30.0	61.5	55.8	39.1	40.7	67.9
	Ostreococcus lucimarinus	36.0	68.4	64.5	43.7	47.3	75.5
	Ostreococcus tauri	35.8	65.0	74.7	42.1	45.4	76.5
	Micromonas sp. RCC299	33.5	66.0	57.4	42.0	44.7	71.7
	Micromonas pusilla	31.0	61.9	52.1	38.4	41.3	67.5
	Bathycoccus prasinos	35.1	68.4	60.1	43.3	45.2	74.6
	Volvox carteri	27.6	54.3	46.6	34.4	36.8	59.6
	Chlamydomonas reinhardtii	25.3	50.6	45.6	32.1	33.8	57.0
	Monoraphidium neglectum	27.1	52.6	47.8	34.0	36.7	60.7
	Coccomyxa subellipsoidea C-169	37.6	66.4	62.4	43.9	48.0	71.5
	Chlorella variabilis	36.5	65.0	61.9	42.6	46.7	71.0
	Auxenochlorella protothecoides	40.8	67.2	65.9	44.5	48.6	73.3
Red algae	*Cyanidioschyzon merolae*	45.7	74.3	68.4	49.0	54.1	78.7
	Galdieria sulphuraria	41.8	71.2	70.1	47.9	52.9	77.5
	Pyropia yezoensis	27.4	52.9	45.7	34.7	36.4	60.2
	Chondrus crispus	30.4	52.3	47.7	36.2	39.0	56.7
	Porphyridium purpureum	35.5	66.3	57.2	43.3	45.5	71.5
Glaucophyceae	*Cyanophora paradoxa*	14.0	31.4	25.7	19.5	19.8	37.0

Oomycetes	*Phytophthora ramorum*	33.8	64.6	53.8	42.8	44.9	69.5
	Phytophthora sojae	27.3	58.6	49.7	38.3	40.3	63.9
	Phytophthora infestans	24.5	52.2	46.0	32.8	31.1	60.4
	Phytophthora capsici	30.5	58.4	50.6	39.1	40.3	64.6
Diatoms	*Phaeodactylum tricornutum*	32.0	62.6	48.7	39.7	42.0	66.9
	Fragilariopsis cylindrus CCMP 1102	19.5	45.8	32.2	28.2	27.5	50.1
	Thalassiosira pseudonana	29.4	60.1	45.7	38.2	39.4	64.1
Other microalgae	*Aureococcus anophagefferens*	39.3	70.7	61.6	44.7	53.7	77.7
	Ectocarpus siliculosus	30.0	57.4	54.1	35.6	39.6	65.4
	Symbiodinium minutum	16.7	42.4	31.5	26.0	27.1	47.2
	Emiliania huxleyi	20.6	52.0	34.0	31.4	31.6	56.5
	Guillardia theta	21.5	48.7	34.4	29.2	30.6	52.6
	Bigelowiella natans	24.8	53.5	41.4	33.3	35.4	58.3
Total of Land plants		34.2	67.9	70.7	44.9	47.4	77.3
Total of Algae		26.9	54.6	46.2	34.7	36.6	60.4
Total of all species		28.0	56.5	49.7	36.2	38.2	62.8

[a] Poor annotations such as "poorly characterized" in KOG, "domain unknown function (DUF)" in Pfam, and "Uncharacterized protein," "Putative uncharacterized," "Unnamed product," and only ID in UniProtKB, were excluded from hits.

[b] The values of total percentage were calculated by combining the results of KOG, Pfam, UniProtKB, GO and PDB.

Table S3. KOG category details

Group	Functional category
Information storage and processing	Translation, ribosomal structure and biogenesis
	RNA processing and modification
	Transcription
	Replication, recombination and repair
	Chromatin structure and dynamics
Cellular processes and signaling	Cell cycle control, cell division, chromosome partitioning
	Nuclear structure
	Defense mechanisms
	Signal transduction mechanisms
	Cell wall/membrane/envelope biogenesis
	Cell motility
	Cytoskeleton
	Extracellular structures
	Intracellular trafficking, secretion, and vesicular transport
	Posttranslational modification, protein turnover, chaperones
Metabolism	Energy production and conversion
	Carbohydrate transport and metabolism
	Amino acid transport and metabolism
	Nucleotide transport and metabolism
	Coenzyme transport and metabolism
	Lipid transport and metabolism
	Inorganic ion transport and metabolism

	Secondary metabolites biosynthesis, transport, and catabolism
Poorly characterized	General function prediction only
	Function unknown

This description is based on the file from “ftp://ftp.ncbi.nih.gov/pub/COG/COG/fun.txt.”
